# Chemokines in Renal Diseases

**DOI:** 10.1100/tsw.2005.105

**Published:** 2005-09-29

**Authors:** Stephan Segerer, Peter J. Nelson

**Affiliations:** Klinikum der Universität, Medizinische Poliklinik-Innenstadt, University of Munich, Pettenkoferstr. 8a, 80336 Munich, Germany

**Keywords:** chemokine receptor, chemokines, glomerulonephritis, renal allograft rejection, chemokine antagonist

## Abstract

The chemokines, members of a large family of chemotactic cytokines, act as directional cues for sorting inflammatory cell subsets to sites of inflammation or lymphoid microenvironments. In addition to their effects on migration, chemokines can also activate effector function in leukocytes and are involved in cell proliferation and angiogenesis. Therefore, it is not surprising that chemokines play important roles in a wide range of human diseases, including genetic immunodeficiencies, infections, autoimmune diseases, and malignant tumors. In this report, we have reviewed recent developments (since mid 2003) in chemokines in renal diseases. In animal models, chemokines are produced at the site of injury, leading to inflammatory cell recruitment. The therapeutic impact of the blockade of CCR1, CCR2, CCR4, CCR5, or the corresponding ligands has been further studied in various renal disease models. Recent studies on the role of the chemokine receptors in human diseases have demonstrated the expression of CXCR1, CXCR3, CCR2, and CCR5 on different subsets of inflammatory cells. The number of CCR5- and CXCR3-positive interstitial infiltrating cells (mainly T cells) correlates with renal function and proteinuria in glomerular diseases. Polymorphisms of chemokines and chemokine receptors are of impact on renal disease courses and allograft survival. Chemokine receptor blockade has approached clinical applications in nonrenal diseases and awaits the application in patients with kidney diseases.

## INTRODUCTION

Inflammatory cells play pivotal roles in renal injury and renal allograft rejection[1,2,3]. The initial insult, which can affect virtually any part of the kidney, activates intrinsic renal cells that respond with the release of proinflammatory mediators. Subsets of inflammatory cells are then recruited to particular renal compartments, e.g., glomerular tufts during glomerulonephritis, arteries during vascular allograft rejection, and the tubulointerstitium in all forms of progressive kidney diseases. These inflammatory cells propagate and amplify the response (through the release of cytokines and growth factors), modulate extracellular matrix synthesis (by the release of proteinases), promote further injury (via reactive oxygen species), and initiate healing and repair (through the clearance of immune complexes or cellular debris)[1,4,5]. In virtually all forms of progressive renal diseases, the tubulointerstitium becomes involved in the injury process. The spillover of proinflammatory mediators, activation of tubular epithelial cells via proteinuria, hypoxia, and release of reactive oxygen species might all result in interstitial inflammation, which finally promotes interstitial fibrosis[6]. The number of interstitial T cells and macrophages seen generally correlates with renal function in the various forms of human glomerular diseases[2,7].

Chemokines are small, chemotactic cytokines that provide a complex signal system for cell communication between all kinds of cells, including leukocytes[8]. Whereas the defining actions are inflammatory cell recruitment and positioning, these cytokines fulfill a variety of biological functions as they modulate angiogenesis and fibrogenesis, as well as proliferation and polarization of hematopoetic progenitor cells[9,10]. During the multistep process of cell recruitment, chemokines act in concert with other soluble mediators, adhesion molecules, and the adhesion molecule counterreceptors[11]. Chemokines need to be presented on endothelial cells to rolling leukocytes, activating integrins and, therefore, promoting firm adhesion[12]. Following extravasation, chemokines guide the way to sites of tissue injury of special microenvironments. This is an important part of immune surveillance, but the same mechanisms are responsible for the recruitment of effector cells during autoimmunity, allograft rejection, and other forms of chronic inflammation, where it is detrimental and leads to tissue damage. It has been hoped that targeting chemokines or their receptors might result in drugs that are more specific (with less side effects) for a defined inflammatory disease[13].

The literature on chemokines in kidney diseases currently focuses on (1) chemokines as therapeutic targets, (2) urinary chemokines and chemokine receptors as diagnostic tools, (3) chemokine polymorphisms as predisposing or prognostic factors, and (4) characterization of chemokines and chemokine receptors in human kidney diseases and allograft rejection.

In this review, we will start with the basics and follow with these topics primarily focusing on what has been published on the various renal diseases since mid 2003 (for detailed reviews of earlier data see [4,14,15,16,17,18]).

## SOME BASIC "CHEMOKINENOLOGY"

The human chemokine family consists of over 40 ligands (L) and 19 corresponding receptors (R)[19]. The subfamilies are grouped according to four conserved cysteine residues in the primary amino acid structure (CCL, CXCL, CX3CL1, and XCL1)[19,20]. Most chemokines bind multiple chemokine receptors of the same subgroup, but chemokines can also act as antagonists for chemokine receptors of other groups. The N terminus is important for receptor binding and specificity, whereas areas in the core and the C terminus are important for multimerization, glycosaminoglycan binding, and presentation of the chemokine on the endothelial surface[21]. Still little is understood about how chemokines are presented in the kidney, which is thought to be mediated by selective glycosaminglycans and most likely by chemokine binding proteins[21,22,23].

Two functionally characterized groups, i.e., inflammatory and homeostatic chemokines, have been described. Inflammatory chemokines like CCL2/MCP-1, CCL5/RANTES, or CXCL10/IP-10 are rapidly upregulated and released by proinflammatory cytokines. Homeostatic chemokines, on the other hand, are involved in tissue homeostasis and cellular recirculation under noninflammatory conditions. The renal literature has focused on inflammatory chemokines, with very little exceptions[24,25,26].

Chemokine receptors are seven, transmembrane-spanning proteins coupled to heterotrimeric G proteins and activate various intracellular pathways like small GTPases and kinases[13]. Chemokine receptors signal on binding chemokines of one class and are therefore labeled accordingly (CCR, CXCR, CX3CR1, XCR1). After ligand binding, chemokine receptors can be rapidly internalized, which stops cell movement and enables response to other chemotactic factors.

A series of seven, transmembrane, chemokine-binding proteins that do not signal in response to binding and are now referred to as interceptors (chemokine-internalizing receptors) are under study[27]. Two members of this family are DARC (the duffy antigen receptor) and D6. DARC binds both CC as well as CXC chemokines[8]. It is expressed on peritubular capillaries in the human kidney and is upregulated during various forms of interstitial injury[28,29]. D6 is expressed on lymphatic endothelial cells, internalized chemokines, which results in chemokine degradation[30,31]. The potential role of these proteins in renal diseases is still unknown.

## CHEMOKINES AS THERAPEUTIC TARGETS

All intrinsic renal cells can express chemokines in response to stress (for details see [4,32]). To better characterize individual chemokines or chemokine receptors as therapeutic targets, a series of mouse models of renal diseases have been employed.

The MRL(lpr/lpr) mouse is a well-defined model of lupus nephritis[33]. A variety of chemokines are upregulated during the disease course (including CCL2/MCP-1 and CCL5/RANTES), associated with the recruitment of inflammatory cells, both to the glomerular tuft and the tubulointerstitium[34]. CCL2/MCP-1–deficient mice demonstrated a significant improvement of the disease course[35]. Deficiency of the corresponding receptor CCR2 resulted in a reduction in the inflammatory cell recruitment (both T cells and macrophages) and improved renal morphology (Perez de Lema et al. [2004], Unpublished data, *J. Am. Soc. Nephrol*. **15,** 686 A). Gene transfer of an NH2-terminal deletion mutant of the MCP-1 gene (7ND) was used to interfere with CCR2 signaling[36,37]. This approach was shown to reduce renal inflammation and prolonged survival[36]. Treatment with the CCR1 antagonist BX471 was found to reduce blood urea nitrogen levels, interstitial T cell and macrophage accumulation, and decreased interstitial fibrosis in this model[33].

An additional approach designed to interfere with chemokine signaling involved the transfection of an N-terminally truncated CX3CL1/fractalkine gene (Fkn-AT) into a fibroblastoid cell line, which was then injected subcutaneously[38]. MRL/lpr mice treated with Fkn-AT before the onset or during the early stages of lupus nephritis demonstrated a reduction of glomerular hypercellularity, glomerulosclerosis, crescent formation, and vasculitis[38]. While these results demonstrate a beneficial effect of chemokine blockade in the MRL-lpr mouse using various approaches, more needs to be learned about the specific chemokine receptor expression by inflammatory cells infiltrating the glomeruli during human lupus nephritis.

The role of macrophage-derived chemokine (CCL22/MDC) and its corresponding receptor (CCR4) has been studied in a nephrotoxic nephritis model in rats[39]. Induction of CCL22/MDC mRNA and protein was detected in nephritic glomeruli. A blockade of CCL22/MDC using specific antibody did not affect the early phase of the disease, but did suppress macrophage recruitment, crescent formation, and deterioration of renal function in the later phase of the disease[39].

Adriamycin nephropathy is a toxic model induced in mice or rats that leads to proteinuria and interstitial fibrosis[40,41]. During the disease course, an increase in expression of chemokines such as CCL5/RANTES and CCL2/MCP-1, and their corresponding receptors, is seen. The induction of autoantibodies directed against CCL5/RANTES and CCL2/MCP-1, achieved through vaccination with naked DNA in rats, resulted in decreased proteinuria, improved creatinine-clearance, conservation of renal morphology, and reduced inflammatory interstitial infiltrates[40]. Accordingly, blocking CCR1 (a receptor for CCL5/RANTES) with a small molecule antagonist decreased interstitial T cell and macrophage accumulation, and reduced interstitial fibroblast accumulation and interstitial fibrosis[41].

To study the role of hepatocyte growth factor (HGF) in renal inflammation, rats with subtotal nephrectomy were treated with recombinant HGF or with a blocking antibody against HGF[42]. HGF treatment reduced tubular CCL2/MCP-1 and CCL5/RANTES expression and decreased inflammatory cell recruitment. Blocking endogenous HGF had the opposite effect[42]. Therefore, the beneficial effects of

HGF in progressive renal injury may be mediated, in part, through reduction of selective chemokine expression.

Unilateral ureter obstruction is commonly used as a rapid model of interstitial fibrosis in mice[43]. CCR1-/- mice demonstrated a significant decrease of interstitial macrophages (by 35%) and lymphocytes (by 55%), as well as reduction of fibroblasts, interstitial volume, and collagen I deposition[44]. In contrast, the disease course was not altered in CCR5-deficient mice as compared to wild type mice. Furthermore, macrophages and T cells from CCR5-deficient mice injected into mice with UUO were efficiently recruited to the obstructed kidney, whereas the number of CCR1-deficient cells or cells treated with a CCR1 antagonist was reduced[44]. Therefore, in UUO of mice, cell recruitment is mainly driven by CCR1 and not by CCR5.

Collagen 4A3–deficient mice develop progressive renal failure, mimicking human Alports syndrome[45]. These mice eventually die from renal failure, which presents with prominent interstitial macrophage accumulation and fibrosis[46]. Treatment of the mice with a CCR1 antagonist resulted in a (BX471) reduced interstitial inflammation, decreased tubular epithelial cell apoptosis, decreased interstitial fibrosis, and prolonged survival[46]. Therefore, there is compelling evidence from a series of murine models of progressive renal injury that blockade of CCR1 shows efficacy.

Ischemia/reperfusion (I/R) injury promotes delayed graft function and is a major obstacle to long-term renal allograft survival. In a rat I/R model (4-h cold ischemia), the induction of CINC (a rat homolog of CXCL8/IL-8) was associated with granulocyte recruitment[47]. Treatment with repertaxin (a small molecule, noncompetitive allosteric inhibitor of CXCR1 and CXCR2[48]) significantly reduced the granulocyte recruitment and serum creatinine. The first dose of repertaxin was given 24 h before surgery.

As with any effective agent, chemokine antagonists may have adverse effects related to specific function of the chemokine in other systems, to unspecific interactions, or organ toxicity. Met-RANTES and AOP-RANTES, two CCL5/RANTES-based functional antagonists, have been found to aggravate glomerular damage and proteinuria in mice with immune complex glomerulonephritis, despite a reduction of glomerular leukocyte infiltration[49]. This unexpected finding may relate to additional biologic roles for the targeted receptors outside leukocyte recruitment. Both functional antagonists inhibit glomerular macrophage recruitment, but also lead to an activation of resident macrophages as characterized by iNOS expression and reduced phagocytic uptake of apoptotic cells, mechanisms that contribute to the repair of glomerular tissues[49]. Apparent exacerbation of renal disease by targeting single chemokine receptors has also been found in mice deficient for the chemokine receptors CCR1 and CCR2[50,51].

## CHARACTERIZATION OF CHEMOKINES AND CHEMOKINE RECEPTORS IN HUMAN KIDNEY DISEASES AND ALLOGRAFT REJECTION

It is now generally accepted that the severity of interstitial injury correlates well with renal function and is of prognostic value for glomerular diseases[5,52]. Recent studies have demonstrated that the general number of interstitial T cells and macrophages correlate with disease outcome. Consistently, the number of chemokine receptor–expressing, infiltrating, inflammatory cells also correlates with the disease severity. The number of interstitial CXCR3 and CCR5 positive cells (which mainly represent the interstitial T cell infiltrate) correlates with renal function and proteinuria at the time of renal biopsy in glomerular diseases[7]. In patients with membranous nephropathy, the number of interstitial, CD68-positive macrophages, CCL2/MCP-1 expression, and the number of interstitial, CCR2-positive cells were significantly higher in initial biopsies in patients that developed end-stage renal disease (ESRD), as compared to those that did not develop ESRD[53]. In crescentic glomerulonephritis, CCR2 was expressed by both glomerular and interstitial macrophages in association with expression of CCL2/MCP-1 by intrinsic renal cells and inflammatory cells[54]. In various forms of glomerulonephritis, the fractalkine receptor CX3CR1 was expressed by T cells in the interstitium and macrophages both in glomeruli and interstitial infiltrates. CXCR1 is a receptor for CXCL8/IL-8, a major chemoattractant for polymorphonuclear leukocytes (PMNs)[20]. The expression of CXCL8/IL-8 has been described in glomerular diseases in association with PMN infiltration[4]. We localized the corresponding receptor, CXCR1, in renal biopsies with glomerular diseases (S. Segerer, unpublished data). CXCR1 was expressed by glomerular PMNs with particularly high numbers in membranoproliferative glomerulonephritis, lupus nephritis, and crescentic glomerulonephritis. Furthermore, CXCR1 is expressed by intrinsic renal cells as smooth muscle cells on the endothelium of a small number of peritubular capillaries and rarely in podocytes. A lot of progress has been made on the expression of chemokine receptors in human diseases, but still a lot of work needs to be done on the role of chemokine receptors during the disease course. Based on early studies of Hancock and colleagues' heart allograft models in the mouse, many subsequent studies have been performed on the potential role of CXCR3 and the corresponding ligands CXCL9/Mig, CXCL10/IP-10, and CXCL11/I-TAC during renal allograft rejection[55]. The studies in CXCR3- and CCR5-deficient mice await confirmation by other groups.

Panzer et al. demonstrated a significant increase of CXCR3-positive cells during acute allograft rejection and a significant induction of the corresponding ligands[56]. In a comparison between humoral and cellular rejection, we demonstrated that only cellular rejection was associated with an accumulation of CXCR3-positive cells in renal allografts[57].

## URINARY CHEMOKINES AND CHEMOKINE RECEPTORS AS DIAGNOSTIC TOOLS

CCL2/MCP-1 mRNA and protein measured in the urine of patients with lupus nephritis correlated with the clinical disease activity and the histological activity score[58]. In a prospective study, urinary CCL2/MCP-1 and CXCL8/IL-8 excretion was quantified in lupus patients[59]. Patients with renal flares demonstrated significantly higher urinary CCL2/MCP-1 excretion as compared to patients with nonrenal flares and healthy controls. Urinary CCL2/MCP-1 excretion correlated with proteinuria, impaired renal function, and was particularly high in patients with proliferative lupus nephritis[59]. Successful treatment decreased urinary CCL2/MCP-1 excretion[59]. Urinary CXCL8/IL-8 was not associated with lupus flares.

As described earlier, CXCR3 and its corresponding ligands are upregulated in patients with cellular renal allograft rejection. Therefore, measuring the corresponding ligands might represent a potential tool for the surveillance of allograft damage. In a baboon model of renal allograft rejection, the urinary excretion of CXCL9/Mig and CXCL10/IP-10 was significantly increased during acute allograft rejection and was found to rise before an increase in serum creatinine was seen[60]. To evaluate urinary excretion of CXCL9/Mig and CXCL10/IP-10 as potential markers of allograft rejection, 69 renal allograft recipients were prospectively evaluated in the early post-transplant period by ELISA[61]. Biopsy-proven allograft rejection was subsequently shown to have developed in 14 patients and rejection was suspected in 1 additional patient. The excretion of CXCL9/Mig was found to be significantly higher in these patients as compared to normal controls, and to allograft recipients without rejection. Urinary CXCL9/Mig excretion successfully predicted acute rejection with a sensitivity of 93% and a specificity of 89%. Importantly, an increase of urinary CXCL9/Mig preceded the clinical diagnosis and was found to decrease after successful antirejection therapy[61]. Tatapudi and colleagues characterized CXCL10/IP-10 and CXCR3 mRNA in urine specimens of renal transplant patients[62]. The authors found a sensitivity of 100% and a specificity of 78% using a cutoff value of 9.11 copies of CXCL10/IP-10, and a sensitivity of 63% and specificity of 83% using the cutoff value of 11.59 copies of CXCR3. Measuring CCXL10/IP-10 in sera from 316 cadaver kidney graft recipients identified serum CXCL10/IP10 levels as a negative predictor for allograft failure[63].

These results build on reports, dating back to the middle of the 90s, that demonstrate that the measurement of chemokines in the urine may represent an important tool in clinical nephrology. The clinical application has not been reached and larger, prospective studies are clearly needed to demonstrate clinical applicability and the impact on disease courses.

## CHEMOKINE POLYMORPHISMS AS PREDISPOSING OR PROGNOSTIC FACTORS

Genetic variations including single nucleotide polymorphisms (SNPs) in chemokines or chemokine receptors have been linked to diverse inflammatory diseases. It is thought that these variations may change the tissue response to injury or the release of chemokines by infiltrating cells.

Several polymorphisms of chemokine and chemokine receptor genes of the host are linked to the course of allograft dysfunction after renal transplantation[55]. An important functional role has been demonstrated for the CCR5 mutation (CCR5Delta32). This mutation results in an absence of functional CCR5 on the cell surface. Patients homozygous for CCR5Delta32 demonstrated a prolonged renal allograft survival[64]. The G allele of the CCL2/MCP-1-2518 promotor polymorphism is associated with an increased CCL2/MCP-1 expression in leukocytes and a decreased renal allograft survival[65]. Significant reductions were found in the risk of acute renal transplant rejection in recipients with the CCR2-64I allele or who were homozygous for the CCR5 59029-A allele[66]. Little is known about the role of donor polymorphisms on the outcome of allografts. It is hoped that with the information of polymorphisms, the immunosuppressive therapy might be tailored to low or high risk groups.

The CCR5Delta32 mutation has also been associated with a better renal outcome in patients with IgA nephropathy[67]. In a study of chemokine polymorphisms in Chinese children with lupus, a -28C/G RANTES/CCL5 promotor polymorphism was associated with increased risk of developing lupus, with higher ANA levels, lower complement, and higher incidence of central nervous system involvement[68].

No association was found with -2518(A/G) MCP-1 or an additional RANTES/CCL5 polymorphism at -403 (G/A) and the course of IgA nephropathy[69].

In a prospective study, Böger et al. included 225 Caucasian patients with type 2 diabetes mellitus on dialysis for less than 2 years[70]. Polymorphisms of CCL5/RANTES (In1.1T/C, -403, and -28), CCL2/MCP-1 (-2518), and CX3CR1 (T280M) were characterized by real-time RT-PCR. A significantly higher risk of all cause mortality was demonstrated in patients with the CCL5/RANTES -403 and the In1.1T/C mutation[70]. Mortality was mainly due to cardiovascular events. Renal diseases are associated with a very high cardiovascular risk, which might even be potentiated by genetic variants of the chemokine system.

## TOLL-LIKE RECEPTORS, CHEMOKINES, AND RENAL DISEASES

Toll-like receptors (TLR) are a family of receptors that bind to pathogen-associated molecular patterns[71]. These receptors help upregulate the expression of proinflammatory cytokines and chemokines in response to LPS (TLR4), lipoproteins and peptidoglycans (TLR 1,2,6), viral RNA (TLR3), and bacterial or viral unmethylated cytosine-guanosin dinucleotide (CpG) DNA (TLR 9)[71]. TLR represent an intricate part of the innate immune response and act as bridge between the innate and adaptive immune response. TLR are expressed on select subsets of inflammatory cells and on intrinsic cells (e.g., tubular epithelial cells). Mouse renal tubular epithelial cells express TLR1, -2, -3, -4, and -6 *in vitro*. Stimulation with LPS leads to an induction of CC chemokines (e.g., CCL2/MCP-1 and CCL5/ RANTES)[72]. This mechanism is thought to mediate the interstitial inflammation seen during sepsis or infection. TLR3 (a receptor for double-stranded RNA, dsRNA) was found to be expressed by interstitial inflammatory cells and mesangial cells in MRL lpr/lpr mice. *In vitro* exposure of mesangial cells to polyinosinic-cytidylic acid (pI:C) RNA led to an induction of CCL2/MCP-1[73,74]. Injection of pI:C RNA into MRL lpr/lpr mice aggravated the lupus nephritis. TLR9 was also localized to glomerular, tubulointerstitial, and perivascular infiltrates in MRL lpr mice, but not intrinsic renal cells[75]. Injection of CpG-oligodeoxynucleotides resulted in an increase in anti-DNA antibodies, an aggravation of crescentic nephritis, interstitial fibrosis, and increased proteinuria[75]. Increased chemokine expression was associated with increased glomerular and interstitial inflammatory cell recruitment. These mechanisms may provide insight into how infections help trigger lupus nephritis flares, via activation of TLR on both inflammatory cells and intrinsic renal cells.

## SUMMARY

Promise of chemokine-based therapeutics in renal disease is beginning to show results. Blockades of select receptors appear to reduce the infiltration of specific leukocytes and thereby limit tissue damage. In human glomerular diseases and allograft rejection, both CCR5 and CXCR3 are currently the most promising targets with CCR5 antagonists demonstrating therapeutic activity in patients with HIV infection and other new drugs in the pipeline (e.g., TAC 220)[76]. In mouse models, CCR1 has been demonstrated to be a suitable target, but the CCR1-expressing cell types have not been described in human renal diseases. While chemokine receptor antagonism may work as a standalone therapy, their use in combination with other anti-inflammatory regimens may be the most efficacious. In addition, the detection of chemokines in urine may prove to be an important early indicator of specific renal damage.

## ACKNOWLEDGMENTS

S.S. is supported by a grant from the Else Kröner-Fresenius Stiftung.
